# Supplementary material for: Patterns of Intron Gain and Loss in Fungi
Source: PLoS Biol. 2004 Nov 30;2(12):e422. doi: 10.1371/journal.pbio.0020422 (PMC532390; doi:10.1371/journal.pbio.0020422)
Supplement: Table S1 — Also available at http://genes.mit.edu/NielsenEtAl/. (4.3 MB ZIP). [file pbio.0020422.st001.zip › NielsenEtAl/html/650.html]

AN4401.1.NCU04303.1.MG00969.1.FG09934.1


```
 CLUSTAL W (1.82) Multiple Sequence Alignments - Introns Inserted


Sequence 1: NCU04303.1	581 aa
Sequence 2: FG09934.1	579 aa
Sequence 3: MG00969.1	583 aa
Sequence 4: AN4401.1	571 aa
Alignment Length: 583 aa
Number Identitical Residues: 398 aa
Alignment Score (without introns) 18133


MG00969.1 	MCGIFACHA2HPDVQKFKPTALRLAKA2IRHRGPDWS1GSVTCNQTI~LCHERLSIVGVE
NCU04303.1	MCGIFACHN2HPDVAKFKPTALKLSKA2IRHRGPDWS1GSVTCHNTI1LCHERLSIVGVE
FG09934.1 	MCGIFACHR2HPDVEKFKPTALKLAKQ2IRHRGPDWS1GSVISNHTI1LCHERLSIVGVE
AN4401.1  	MCGIFACHH2HPDVQAFKPTALRMAKA2VRHRGPDWS1GSFIADKTI1LCHERLCIVGVD
          	********  ****  ******:::*  :******** **. ..:** ******.****:

MG00969.1 	1SGAQPLTNEDESIILAVNGEIYNHRLVRKSLKTPYHFKTTSDCEVIIPL0YMEHGLDAP
NCU04303.1	1SGAQPLTNADESIAVAANGEIYNHRLIRKHLKNPYHFKTTSDCEVIIPL0YLEHGVDAP
FG09934.1 	1SGAQPLTNADDSIILAVNGEIYNHRHIRKHLKERYHFKTTSDCEVIIPL0YTEFDTDAP
AN4401.1  	1SGAQPLVNDDESLALAVNGEIYNHRIVRKTLDVEYNYKTHSDCEVIIPL0YMKYGLDAP
          	 ******.* *:*: :*.******** :** *.  *::** ********* * :.. ***

MG00969.1 	KYLDGMFSFVLYDKKQNRTIAARDPIGVTTFYQGWSSSEPGAVYFGSELKCLYQVCDKIE
NCU04303.1	KHLDGMFSFVLYDKKQDRTIAARDPIGITTLYKGYNSQEPGTVYFASELKSLHPVCDKIE
FG09934.1 	NHLDGMFSFVLYDKKQDRTIAARDPIGITTFYQGWSSTEPGTIYFASELKCLHSVCDKIV
AN4401.1  	KHLDGMFAWVLYDKKEDRVIAARDPIGICSFYIGWSSQTPGAVYFASELKSLHPVCDKVE
          	::*****::******::*.********: ::* *:.*  **::**.****.*: ****: 

MG00969.1 	AFPPGHIYDSLTGERTRYFQPTWWDPKRVPETPLDLTKLREALEKSVRKRLMAEVPYGVL
NCU04303.1	AFPPGHVFDSLTGETTRYFEPSWWDEKKCPQTPLDLKLLRETLEKSVRKRLMAEVPYGVL
FG09934.1 	AFPPGHVYDSKTGETTRYFNPSWWDPKKVPSTPVDYKVLRQALEKSVRKRLMAEVPFGVL
AN4401.1  	AFPPGHVFDSKSGEFSRYFQPSWWDPTNVPSTPVDYKAIRESLTRSVRKRLMAEVPYGVL
          	******::** :** :***:*:*** .. *.**:* . :*::* :***********:***

MG00969.1 	LSGGLDSSLVASIAQRETKRLKKLAIEAGLEDLPAEPTGNHDQGEGLVGIDDENKLSTMT
NCU04303.1	LSGGLDSSLVAAIAQRESLRLRKLAEEQAALNQEVDPE-AQDKGEGLVGIDDENKLSTVT
FG09934.1 	LSGGLDSSLTASIAQREVTRLRKQALEANGN--VLPVE-DPDTGEGLVGVDDDDHLDTLT
AN4401.1  	LSGGLDSSLVASIAQRETLRMQKASSG-----------VKSEANGELVGIDDENELSTVN
          	*********.*:*****  *::* :                : .  ***:**::.*.*:.

MG00969.1 	YLPQLNSFSIGLPGSPDNKAALEVAKFLGTKHHVMTFTIEDGLNALSDVIYHLESYDVTT
NCU04303.1	FLPQLNSFSIGLPGSPDNKAALEVAKFLGTKHHVMTFTIEDGLNALSDVIYHLETYDVTT
FG09934.1 	YLPQLNSFSIGLPGSPDNKAALEVAKFLGTKHHVMTFTIEDGLNALSDVIFHLETYDVTT
AN4401.1  	SFQQLQSFSIGLPGAPDTEAAMEVAKFLGTKHHALTFTIEDGLNALSDVIYHLETYDVTT
          	 : **:********:**.:**:***********.:***************:***:*****

MG00969.1 	IRASTPMYLLSRKIKAMGIKMVLSGEGSDEAFGGYLYFHNAPDKDAFHDETVRRVKNLHL
NCU04303.1	IRASTPMYLLSRKIKAMGIKMVLSGEGSDEIFGGYLYFHGAPNKEEFHEECVRRVKNLHL
FG09934.1 	IRASTPMYLLSRKIKAMGIKMVLSGEGSDEIFGGYLYFHAAPDKDSFHEETVRRVKNLHL
AN4401.1  	IRASTPMYLLSRKIKGLGVKMVLSGEGSDEIFGGYLYFHAAPNKEEFHKETVRRVKNLHL
          	***************.:*:*********** ******** **:*: **.* *********

MG00969.1 	SDCLRANKSTSAWGLEARVPFLDKE~FLELAMNIDPKEKMITKERIEKYIVRKAFDTSDD
NCU04303.1	ADCLRANKSTSAWGLEARVPFLDKE~FLEVALNIDPQEKMITKEKLEKYILRKAFDTKDQ
FG09934.1 	SDCLRANKSTSAWGLEARVPFLDKE0FLEYSMNIDPQDKMITKEKLEKYILRKAFDTSDE
AN4401.1  	ADCLRANKSTSAWGLEARVPFLDKA~FIETSMSVDPQEKMITKDRIEKYILRKAFDTSDE
          	:***********************  *:* ::.:**::*****:::****:******.*:

MG00969.1 	PNAEPYLPDNILWRQKEQFSDGVGYGWIDALKDNAEIQVTDEMMKNPKPEWGDDIPDTKE
NCU04303.1	P-EGAYLPDNILYRQKEQFSDGVGYGWIDALKDQAELQVTDEMMKNPKPEWGDDIPDTKE
FG09934.1 	PGEQPYLPDNILWRQKEQFSDGVGYGWIDALKDTAEKQVTDEMMKNPKPEWGNDIPDTKE
AN4401.1  	PDVEPYLPDKILWRQKEQFSDGVGYSWIDGLKDHAELHVTDEMMKNPKPEWGDDIPTTKE
          	*.  .****:**:************.***.*** ** :**************:*** ***

MG00969.1 	A~YWYRCMFDEHFPPHCASTVERWTPTWSKQTDPSGR2AIAVHAAKYDHISE
NCU04303.1	A~YWYRCMFDEHFPPSCASTVMRWTPKWVKQTDPSGR~AISIHQAKYDHVDE
FG09934.1 	A~YWYRCMFDEHFPPHCASTVMRWTPTWSKQTDPSGR2AIATHNAKYDSAA-
AN4401.1  	A2YWYRMMFDEHFPSYCASTVERWVPTWSKQSDPSGR2AIATHNAKYDDAE-
          	* **** *******. ***** **.*.* **:***** **: * ****
```
